# Highly Responsive and Ultrasensitive **Non-Enzymatic Electrochemical** Glucose Sensor Based on Au Foam

**DOI:** 10.3390/s19051203

**Published:** 2019-03-09

**Authors:** Nannan Shen, Haijun Xu, Weichen Zhao, Yongmei Zhao, Xin Zhang

**Affiliations:** 1 Beijing Bioprocess Key Laboratory, Beijing University of Chemical Technology, Beijing 100029, China; shen_frank@126.com (N.S.); 2016200913@mail.buct.edu.cn (W.Z.); 2 College of Science, Beijing University of Chemical Technology, Beijing 100029, China; 3 Engineering Research Center for Semiconductor Integrated Technology, Institute of Semiconductors, Chinese Academy of Sciences, Beijing 100083, China; ymzhao@semi.ac.cn

**Keywords:** Au foam, bubble-templated electrodeposition, non-enzymatic glucose sensor, amperometric response

## Abstract

Glucose concentration is an important physiological index, therefore methods for sensitive detection of glucose are important. In this study, Au foam was prepared by electrodeposition with a dynamic gas template on an Au nanoparticle/Si substrate. The Au foam showed ultrasensitivity, high selectivity, and long-term stability in the quantitative detection of glucose. The foam was used as an electrode, and the amperometric response indicated excellent catalytic activity in glucose oxidation, with a linear response across the concentration range 0.5 μM to 12 mM, and a limit of detection of 0.14 μM. High selectivity for interfering molecules at six times the normal level and long-term stability for 30 days were obtained. The results for electrochemical detection with Au foam of glucose in human serum were consistent with those obtained with a sensor based on surface-enhanced Raman spectroscopy and a commercial sensor. This proves that this method can be used with real samples. These results show that Au foam has great potential for use as a non-enzymatic glucose sensor.

## 1. Introduction

Diabetes is a metabolic disease, the main symptom of which is hyperglycemia. Long-term hyperglycemia leads to chronic damage and dysfunction of various tissues, especially the eyes, kidneys, heart, blood vessels, and nerves [1]. The latest report from the International Diabetes Federation states that about 425 million adults worldwide were suffering from diabetes in 2017 and the number has continued to grow. Diabetes is mainly diagnosed by determining the concentration of glucose in blood or urine. The monitoring of glucose in human blood helps diabetics to understand their condition and reduce the incidence of diabetic complications [2,3]. There has also been a growing demand for glucose monitoring in the fermentation [4], food [5] and chemical industries [6] during the past few decades. The development of sensors for fast, sensitive, accurate, and reproducible glucose determination is therefore needed. Many strategies for glucose detection have been developed, including optical [7], fluorescence [8], electrochemical [9], and chromatographic techniques [10]. Electrochemical methods are considered to be the most effective because of their high sensitivity, convenience, speed, and low-cost [11]. Both enzyme-based and enzyme-free electrochemical sensors have been developed [2,11]. In enzymatic electrochemical glucose sensors, glucose oxidase or glucose dehydrogenase interacts with glucose, which leads to charge transfer to the electrode surface, therefore, good selectivity is achieved [12,13]. However, poor stability is an unavoidable problem and greatly decreases the long-term accuracy of enzymatic electrochemical glucose sensors because the enzymatic activity is easily affected by environmental conditions, such as the temperature and pH value [14,15]. Non-enzymatic electrochemical glucose sensors are clearly a better choice because glucose is directly oxidized without the participation of enzymes [16]. The development of non-enzymatic electrochemical glucose sensors with high sensitivity, good stability, reproducibility, and strong anti-interference properties is therefore attracting increasing attention. 

The electrochemical activities of various nanomaterials have been investigated for use in non-enzymatic electrochemical glucose sensors with high sensitivity, selectivity, and long-term stability; these materials include copper oxide [17], ferrite [18], nickel hydroxide [19], Ni foam [20], and carbon nanotubes [21]. However, the high overpotential of glucose oxidation is a problem with these materials. Especially for Ni foam, where the foam structure helps to adsorb a large number of detection molecules, but the limit of detection is high and the detection range is not wide enough. The use of Au to solve this problem has aroused our interest because of its high catalytic activity and excellent biocompatibility [22]. Au nanoparticles were attached to graphene to make glucose sensors, while the small response limited its application [23]. Soomro et al. used Au nanocages to produce a glucose sensor [24], but the sensitivity still needs to be improved. The sensitivity of a glucose sensor is largely limited by the catalytic activity of material, or in the other words, the catalytic activity of the Au nanomaterial contributes directly to the assay performance of a glucose sensor. In the fields of electrochemical oxidation of formic acid and methanol, gold electrodes are often foamed so that they have a three-dimensional porous structure and thus large specific surface areas [25]. The structural characteristic of Au foam enables adsorption of large amounts of substance molecules, and the high active surface area also facilitates electrochemical reactions [26]. It is reasonable to believe that this excellent characteristic can also play a great advantage for the catalysis of glucose oxidization in a non-enzymatic glucose sensor. 

In this paper, we report the study on Au foam for use as a non-enzymatic electrochemical glucose sensor. The foam was prepared by cathodic deposition with a bubble dynamic template [27]. The developed electrode was tested in the amperometric detection of glucose at various concentrations, and its selectivity and stability were assessed. The use of Au foam for the detection of glucose in human serum was also investigated. The results show that the developed non-enzymatic electrochemical glucose sensor has a high sensitivity and selectivity. 

## 2. Materials and Methods

### 2.1. Chemicals and Reagents

Glucose, ascorbic acid (AA), uric acid (UA), acetaminophen (AP), and dopamine (DA), HAuCl_4_, AgNO_3_ were purchased from the Aladdin Industrial Company. H_2_SO_4_, NH_4_Cl, NaOH, and H_2_O_2_ were purchased from Beijing Chemical Works. The human serum was purchased from Beijing Lablead Biotechnology Co., Ltd. The deionized water (18 MΩ) was supplied by Beijing Chemical Works and used for all experiments. All chemicals were of analytical grade and used without further purification. 

### 2.2. Preparation of Au Nanoparticle/Si Substrate

The Au/Si substrate was prepared via the method reported by Zhang et al. [28]. The synthesis was performed at room temperature. N-type (100) Si wafers (1–10 Ω·cm) were used as the substrate material and cut into pieces of dimensions 0.5 cm × 3 cm. The Si wafer pieces were cleaned sequentially in deionized water, acetone, and ethanol, for 5 min each. The wafer was immersed in a solution of 4.6 M HF and 0.005 M AgNO_3_ for 2 min and then washed several times with deionized water. The template was placed in a mixture of 4.6 M HF and 0.5 M H_2_O_2_ for 60 min, and then immersed in a solution of 0.01 M HAuCl_4_ for 30 min. The as-prepared Au nanoparticle (NP)/Si would provide a conductive substrate and anchor points for the subsequent electrodeposition of the Au foam film [29].

### 2.3. Electrodeposition of Au Foam

Electrochemical experiments were performed with a CHI 660C electrochemical workstation (Chen Hua Instruments, Shanghai, China). The Au/Si substrate, platinum foil (geometric area 1 cm^2^), and a saturated calomel electrode were used as the working, counter, and reference electrodes, respectively. Nanoporous Au foam was formed by electrodeposition from an aqueous electrolyte consisting of 0.1 M HAuCl_4_ and 2 M NH_4_Cl. Au foam was deposited potentiostatically by applying a potential of −4 V to the Au/Si electrode. The deposition time was 20 s.

### 2.4. Instrumentations

The micro- and nano-scale morphologies were examined by scanning electron microscopy (SEM; Hitachi S4700). The structural properties were investigated by X-ray diffraction (XRD; Rigaku, CuKα, *λ* = 1.5418 Å). The chemical composition was determined by energy-dispersive X-ray spectroscopy (EDS) in conjunction with SEM. Surface-enhanced Raman spectroscopy was performed at room temperature with a Lab-RAM ARAMIS Raman system. 

## 3. Results and Discussion

### 3.1. Characterizations of Electrodeposited Au Foam

**Figure 1:**
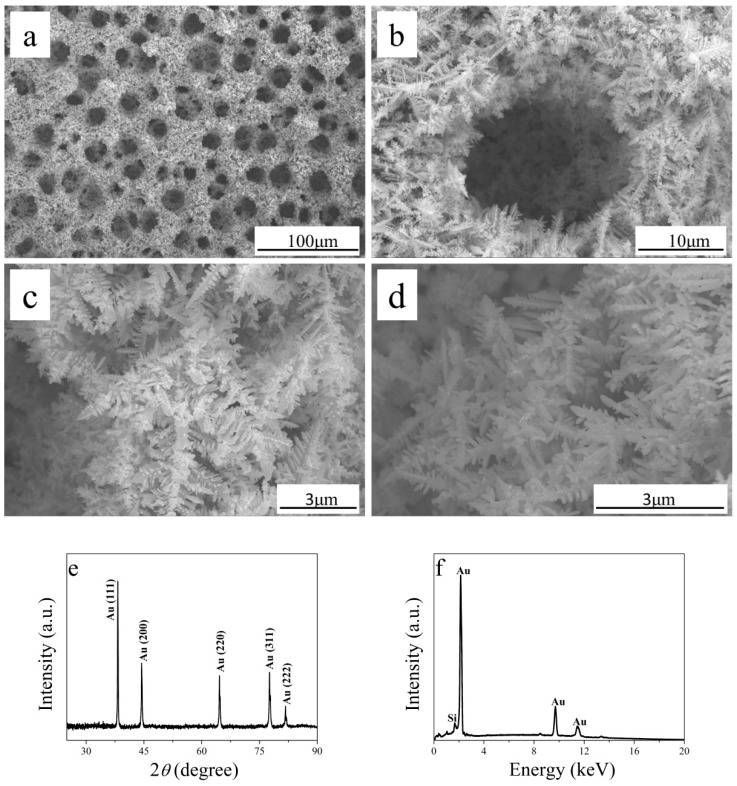
SEM images of (**a**) Au foam, (**b**) magnified view of (**a**), (**c**) pore wall, and (**d**) inner pore; (**e**) XRD pattern and (**f**) energy-dispersive X-ray spectroscopy (EDS) spectrum of Au foam.

SEM was used to examine the surface morphologies and microstructures of the obtained Au foam samples. In the process of the electrodeposition of Au, the highly negative potential of −4 V ensured the production of dense bubbles, which provided a dynamic template for Au deposition [30]. Specifically, the H_2_ and NH_3_ generated by H_2_SO_4_ and NH_4_Cl at a high overpotential formed bubbles on the surface of the Au/Si substrate, and the bubbles gradually became larger, before becoming detached from the surface. At the same time, Au was reduced and dendrites grew, with the formation of a foam structure. Figure 1a shows that the Au foam had a three-dimensional honeycomb structure containing a large number of uniform pores. The Au foam, therefore, had a large surface area and a large number of active sites for glucose oxidation. The magnified image of a pore in Figure 1b shows that the wall consisted of dendrites. Figure 1c shows the pore wall morphology. Au dendrites also formed inside the pores (Figure 1d). The XRD pattern (Figure 1e) of the Au foam shows peaks at 38.2°, 44.6°, 64.6°, 77.6°, and 81.7°, which corresponded to the (111), (200), (220), (311), and (222) crystal faces, respectively, of Au (JCPDS 04-0784). The diffraction peaks from Si were not present, which indicated that Au was well deposited on the Au/Si substrate. The elemental composition of the porous structure was determined by EDS. Figure 1f shows that the peaks from Au were much stronger than those from Si, which shows that a dense Au layer covered the Au/Si substrate. These results clearly show the successful fabrication of Au foam. 

### 3.2. Electrochemical Performance of Au Foam 

**Figure 2:**
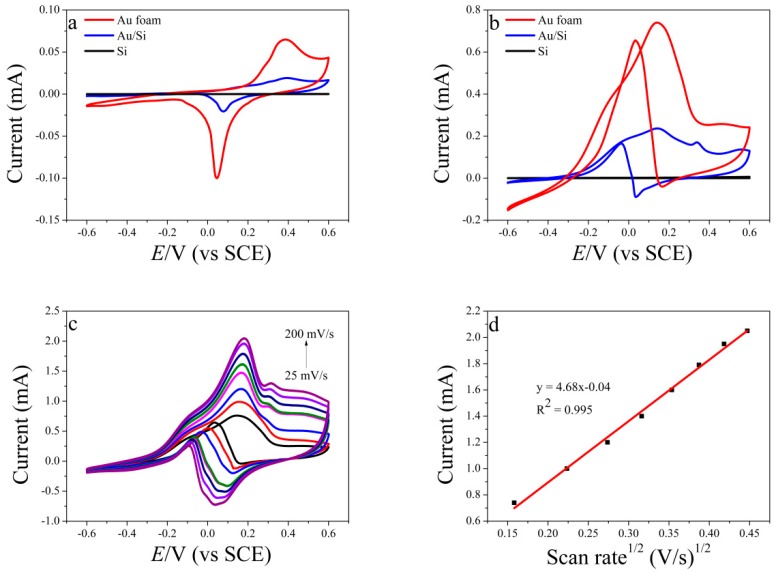
(**a**) Cyclic voltammetry (CV) curves for Au foam, Au/Si, and Si in 0.3 M NaOH at scan rate of 25 mV·s^−1^, (**b**) CV curves for glucose oxidation on Au foam, Au/Si, and Si in 0.3 M NaOH containing 5 mM glucose at scan rate of 25 mV·s^−1^, (**c**) CV curves of Au foam at various scan rates, (**d**) plot of peak current vs. square root of scan rate.

The electrochemical behaviors of the Au foam, Au/Si, and Si electrodes in 0.3 M NaOH solution were studied by cyclic voltammetry (CV). Figure 2a shows that the Au foam gave an anodic peak at 0.38 V, corresponding to the formation of gold oxides such as Au_2_O_3_. A sharp cathodic peak at 0.04 V from the reduction of gold oxides was also observed. For the Au/Si electrode, the redox peak positions shifted slightly from those for the Au foam. The peak current for the Au foam was much larger than that for the Au/Si substrate, which indicated that the electrochemical activity of the Au foam was better than that of Au/Si because of the large surface area of the Au foam. This large surface area was a result of the porous structure and contributed to electrochemical catalysis and detection. Si did not give any peaks. The electrocatalytic activities of the Au foam, Au/Si, and Si in glucose oxidation were compared by performing CV in 0.3 M NaOH with 5 mM glucose at a scan rate of 25 mV·s^−1^. Figure 2b shows that the response of the Au foam to glucose oxidation was stronger than that of Au/Si. This indicated that the electrochemical activity of the Au foam was better than that of Au/Si. During the positive scan, glucose was oxidized to dehydrogenated glucose at −0.1 V [31], and dehydrogenated glucose was further oxidized to gluconolactone, which occurred at +0.2 V. The activity of glucose oxidation on the Au surface, especially via the influence of the adsorbed OH^−^ anions and the gold oxide surface monolayer formation, depended on the surface area and structure [32]. Thus, the exposure of more AuNPs on the Au foam with the large surface area could help enhance the catalytic activity for the electrochemical oxidation of glucose. In the reverse scan, the gold oxides were reduced and then formed AuOH with OH^−^ [33]. Previous studies have shown that AuOH is the active substance in glucose oxidation [34]. There was, therefore, a strong oxidation peak for the oxidization of glucose at around 0.0 V. The catalytic activity of Si in glucose oxidation was excluded on the basis of the CV curve for Si in the glucose solution. No redox peaks from Si were observed for glucose oxidation. 

The electrokinetics of glucose oxidation was investigated by performing glucose oxidation at various scanning rates from 25 to 200 mV·s^−1^ in 0.3 M NaOH solution, with a glucose concentration of 5 mM. The glucose oxidation peak current increased with increasing scan rate (Figure 2c). The square root of the scan rate was proportional to the maximum value of the glucose oxidation peak, with a correlation coefficient of 0.995. This showed that the oxidation of glucose on the Au foam electrode was a diffusion-controlled process. A diffusion control process means that the rate of the electrochemical reaction is higher than that of diffusion, which is beneficial to the application.

### 3.3. Use of Au Foam in Electrochemical Detection of Glucose 

**Figure 3:**
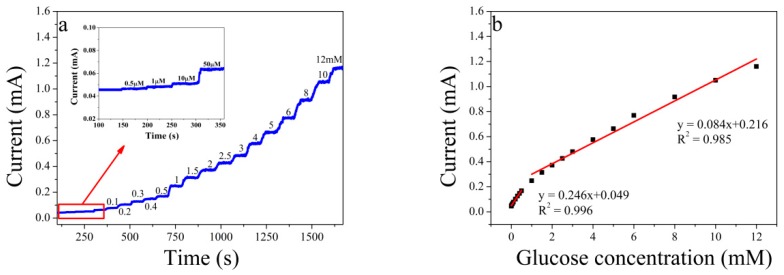
(**a**) Amperometric current–time (*i*–*t*) curves for Au foam at various glucose concentrations at 0.1 V and at intervals of 50 s, and (**b**) plot of glucose concentration vs. current.

The quantitative detection of glucose with Au foam was investigated amperometrically by plotting current–time (*i*–*t*) curves for various concentrations of glucose at a constant voltage. The applied potential significantly affects the sensitivity and selectivity of a glucose sensor. When the potential is less than 0 V, the current intensity increases with increasing glucose concentration first, but it will decrease rapidly if the glucose concentration continues to increase because of the incomplete oxidation of glucose [35]. Therefore, the potential should be larger than 0 V to ensure sensitivity. However, if the applied potential is too high, the selectivity will be affected. This effect is caused by interference from species in human serum, such as uric acid, ascorbic acid, dopamine, and acetaminophen, which are oxidized at the electrode surface at an applied potential of 0.2 V [36]. A potential of 0.1 V was therefore chosen for determining the amperometric responses at the different concentrations of glucose. At this applied voltage, various concentrations of glucose were injected under stirring into 0.3 M NaOH solution at 50 s intervals. Figure 3a shows the *i*–*t* curves for the amperometric responses. The figure shows that when glucose was injected, the current increased. The two linear ranges, as shown in Figure 3b, can be identified in Figure 3a. For the range of 0.5 μM to 1 mM, the linear regression equation is *i* (mA) = 0.246 × *C*_[glucose]_ (mM) + 0.049 (*R*^2^ = 0.996), and for the range of 1 to 12 mM, the equation is *i* (mA) = 0.084 × *C*_[glucose]_ (mM) + 0.216 (*R*^2^ = 0.985). The sensitivity of the sensor was also examined. According to the definition of the International Union of Pure and Applied Chemistry (IUPAC) [37], the limit of detection (LOD) is estimated to be 3 times the signal-to-noise ratio. 3σ, where σ is the standard deviation of seven independent measurements for a blank sample, was introduced into the linear relationship equation for the low concentration region (*i* (mA) = 0.246 × *C*_[glucose]_ (mM) + 0.049) to obtain the detection limit of glucose. The results of the seven blank measurements were 0.04703, 0.06548, 0.02509, 0.05956, 0.03348, 0.02758, 0.05661 mA, giving σ = 0.016345, so the LOD was determined to be 0.14 μM. This LOD was lower than those for most other sensors because of the large surface area, and abundant active sites on the Au foam. 

The activity of the electrode surface at a low glucose concentration differed from that at a high glucose concentration [38]. At low concentrations, there were high numbers of active sites (in relation to the total number of analyte molecules), therefore the response increased more rapidly with increasing concentration. At high glucose concentrations, glucose adsorbed on the electrode covers the active surface, therefore the response increased more slowly.

### 3.4. Selectivity and Long-Term Stability of Au Foam

**Figure 4:**
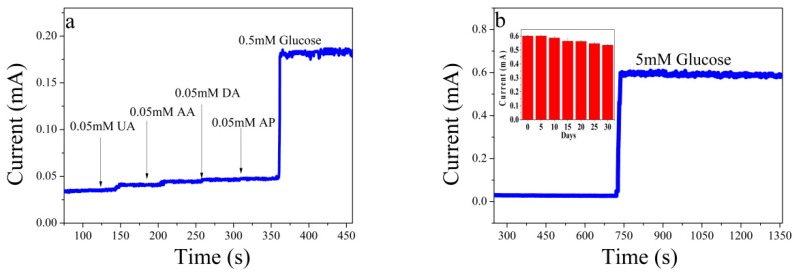
(**a**) Selectivity for glucose (0.5 mM) in presence of uric acid (0.05 mM), ascorbic acid (0.05 mM), dopamine (0.05 mM), and acetaminophen (0.05 mM), and (**b**) amperometric response of Au foam to 5 mM glucose in 0.3 M NaOH solution for running time of 10 min (inset: long-term stability of Au foam with 5 mM glucose for 30 days).

Glucose sensors are mainly used for detection of glucose, which is the main sugar in human blood, therefore it is important to evaluate the effects of small molecules such as UA, AA, DA, and AP in human serum. These substances are co-oxidized at similar potentials, producing noise anodic current and also reducing the sensitivity of the biosensor [39]. The normal concentrations of AA, UA, AP, and DA in the human body are about 0.1 mM [18], and the normal concentration of glucose in the human body is 3.9–6.1 mM. Here, to simulate the internal environment of the human body and amplify the effects of small molecules, we used 0.05 mM AA, UA, AP, and DA, and 0.5 mM glucose in an evaluation of the selectivity of the glucose sensor. Figure 4a shows that the amperometric responses of AA, UA, AP, DA in 0.3 M NaOH, at a potential of 0.1 V, were negligible compared with that of glucose. This shows that the Au foam had excellent selectivity for glucose.

For glucose sensors, the stability of long-term detection is also a key requirement. Figure 4b shows that 97% of the amperometric response of 5 mM glucose at a potential of 0.1 V was retained after running for 10 min. The stability of the Au foam electrode was also determined by measuring the current for 5 mM glucose at a potential of 0.1 V for 30 days. The inset in Figure 4b shows that after 30 days, 90% of the amperometric response was retained. These results showed that, unlike traditional enzymatic glucose sensors, which have poor stability, Au foam has sufficient stability for use as a glucose sensor.

### 3.5. Detection of Glucose in Human Serum

**Figure 5:**
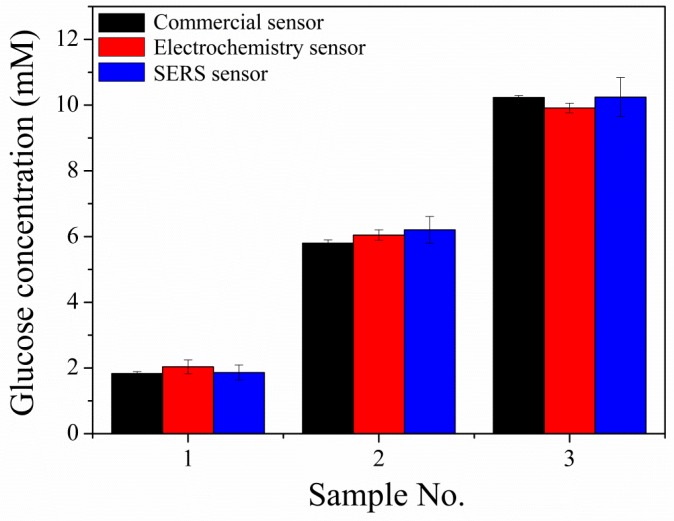
Determination of glucose at different concentrations in alkalized human serum samples with a commercial sensor, electrochemical sensor, and Surface Enhanced Raman Scattering (SERS).

The applicability of Au foam as an electrochemical glucose sensor was further investigated by comparing the results from a commercial sensor, our electrochemical sensor, and our previously reported Surface Enhanced Raman Scattering (SERS) sensor for detection of various concentrations of glucose in human serum. The commercial sensor used was the Blood Glucose Meter 580 from Shanghai Yuyue Medical Equipment Co., Ltd. Following the instrument instructions, we detected the concentration of glucose by dropping the solution on a test paper and then directly reading the concentration of glucose. For the detection using the SERS sensor, detailed information about the SERS sensor and the test method from an article previously published by our group [40] can be referred to. The amperometric responses of three samples were evaluated with our electrochemical sensor at an applied potential of 0.1 V in human serum containing 0.3 M NaOH. We also used a commercial sensor and our SERS sensor for the detection of glucose in the three samples under the same conditions, to assess the reliability of our electrochemical sensor. Figure 5 shows that the responses of our electrochemical sensor and the SERS sensor were in the ranges of 97% to 102% and 93% to 104%, respectively, compared with those of the commercial sensor. This showed that the electrochemical sensor was as precise as the commercial sensor, and confirmed that it has a potential practical application in real sample systems.

## 4. Conclusions

In this study, highly porous Au foam was obtained by electrochemical deposition with an artful hydrogen and ammonia bubble dynamic template. The porous structure gave a large surface area, which contributed to the electrochemical oxidation of glucose. The Au foam showed good electrochemical activity in the detection of glucose, and therefore has potential applications as a glucose sensor. The amperometric responses to glucose at various concentrations were detected electrochemically with excellent sensitivity. The selectivity and stability of the glucose sensor met the requirements for practical applications. In addition, the results of tests on human serum samples suggested good potential for use with real samples. We believe that Au foam has the potential for use as a glucose sensor with excellent sensitivity, selectivity, and long-term stability.
